# Sex and age-specific gamma-glutamyl transferase to high-density lipoprotein cholesterol ratio: a potential marker of subclinical arterial stiffness elevation

**DOI:** 10.3389/fcvm.2026.1852845

**Published:** 2026-08-11

**Authors:** Yi-Chih Chang, Ting-Yu Lin, Wen-Cheng Li

**Affiliations:** 1 Department of Cardiology, Xiamen Chang Gung Hospital, Xiamen, China; 2 Department of Medical Education, Chang Gung Memorial Hospital at Linkou, Taoyuan, Taiwan; 3 Department of Family Medicine, Linkou Chang Gung Memorial Hospital, Taoyuan, Taiwan; 4 College of Medicine, Chang Gung University, Taoyuan, Taiwan

**Keywords:** arterial stiffness, brachial-ankle pulse wave velocity, cardiovascular risk, gamma-glutamyl transferase, GGT/HDL-C ratio, HDL-C, subclinical arterial stiffness elevation

## Abstract

**Introduction:**

The gamma-glutamyl transferase to high-density lipoprotein cholesterol ratio (GGT/HDL-C) integrates oxidative stress and lipid dysregulation, but its relationship with subclinical arterial stiffness elevation (SASE) and potential sex- and age-specific differences remains unclear. This study examined the association between GGT/HDL-C and SASE, assessed using brachial-ankle pulse wave velocity (baPWV), in an asymptomatic adult population.

**Methods:**

This cross-sectional study included 10,518 adults who underwent routine self-paid health examinations at Xiamen Chang Gung Hospital between 2013 and 2016. Clinical, biochemical, and vascular parameters were assessed. SASE was defined as a mean baPWV >1,700 cm/s. Participants were stratified by sex and age (≤50 and >50 years) and categorized into sex- and age-specific tertiles of GGT/HDL-C. Hierarchical logistic regression models were adjusted for age, fasting glucose, triglycerides, low-density lipoprotein cholesterol, alcohol intake, and lipid-lowering medication use. Receiver operating characteristic analyses were performed to evaluate discriminatory performance.

**Results:**

The study included 5,877 men and 4,641 women, with a median age of 46 years; 799 participants (7.6%) had SASE. Higher GGT/HDL-C tertiles were associated with less favorable cardiometabolic profiles and progressively higher baPWV. In the fully adjusted model, the highest versus lowest GGT/HDL-C tertile was associated with increased odds of SASE in men >50 years (odds ratio [OR], 1.823; 95% confidence interval [CI], 1.303–2.550), women ≤50 years (OR, 4.620; 95% CI, 1.012–21.101), and women >50 years (OR, 1.435; 95% CI, 1.009–2.040). In men ≤50 years, the association was positive but of borderline statistical significance (OR, 1.877; 95% CI, 0.994–3.544). Sensitivity analyses yielded generally consistent findings. GGT/HDL-C alone demonstrated modest discriminatory performance, which was better in women than in men.

**Discussion:**

Higher GGT/HDL-C ratios were associated with SASE, with variations in the magnitude and statistical precision of the associations across sex and age groups. The GGT/HDL-C ratio may serve as an adjunctive biomarker for preliminary vascular risk stratification. However, its modest stand-alone discriminatory performance and the cross-sectional, single-center study design warrant cautious interpretation and external validation.

## 1 Introduction

Subclinical atherosclerosis (SA) represents an early, asymptomatic phase of arterial wall disease characterized by structural changes such as intima-media thickening, coronary artery calcification, or the formation of atherosclerotic plaques, detectable through non-invasive imaging modalities. The underlying pathophysiology of SA involves endothelial dysfunction, lipid accumulation, vascular inflammation, and smooth muscle cell proliferation—hallmarks of atherogenesis that precede luminal obstruction or ischemic events (1).

The global burden of SA is significant, particularly among middle-aged and older adults. For instance, a Danish study utilizing multimodal non-invasive screening found that more than half (56%) of apparently healthy individuals aged 50–60 exhibited signs of atherosclerosis in the coronary or carotid arteries (2). Similarly, the Cardiovascular Health Study in the United States revealed a prevalence of 36%–39% in adults over 65 years (3). Importantly, SA is not merely a benign structural change but is closely linked to future cardiovascular morbidity and mortality. In older adults, coronary calcium burden has been shown to correlate with both prevalent and incident CVD, supporting its use as a predictive biomarker (1, 4).

Gamma-glutamyl transferase (GGT) is a membrane-bound enzyme that plays a central role in glutathione metabolism and oxidative stress regulation. High-density lipoprotein cholesterol (HDL-C), conversely, is well recognized for its protective role in cardiovascular health through reverse cholesterol transport and anti-inflammatory effects. The ratio of GGT to HDL-C (GGT/HDL-C) has recently emerged as a novel biomarker that integrates the oxidative and lipid-based aspects of metabolic dysfunction (5), demonstrating superior discriminatory ability for cardiometabolic risk compared to individual components. This composite index has been proposed as a more sensitive indicator of cardiometabolic risk than either marker alone.

GGT is not only a marker of liver function but also a surrogate for oxidative stress, which contributes to the pathogenesis of atherosclerosis. Elevated GGT levels have been associated with increased cardiovascular mortality and coronary artery obstruction severity (6). Concurrently, reduced HDL-C levels are known to impair endothelial function and increase CVD risk. The GGT/HDL-C ratio thus represents a dual-risk profile that reflects both increased oxidative stress and diminished lipid-mediated protection. In Korean populations, a higher GGT/HDL-C ratio was associated with incident cardiovascular disease, with the strongest predictive value observed among women (7). Similar findings were observed in postmenopausal women, where each unit increase in the GGT/HDL-C ratio increased the odds of intermediate or high CVD risk by 10% (8).

Pulse wave velocity (PWV), a non-invasive surrogate of arterial stiffness, quantifies the speed of pressure wave propagation through the arterial system. An elevated PWV reflects reduced arterial compliance and is an independent potential marker of cardiovascular events and all-cause mortality. The most widely accepted standard for central arterial stiffness measurement is carotid-femoral pulse wave velocity (cfPWV), which reflects aortic stiffness. cfPWV is strongly associated with vascular calcification, coronary artery disease, and cardiovascular mortality (9). However, practical limitations such as the need for femoral access and operator dependence hinder its widespread use in routine clinical practice. brachial-ankle pulse wave velocity (baPWV) offers a more accessible and user-friendly alternative, especially in community and outpatient settings. It can be measured quickly using automated oscillometric devices. Although baPWV traverses both central and peripheral arteries, it shows strong correlation with cfPWV and is also predictive of cardiovascular outcomes (10), including stroke and coronary artery disease (11). Studies confirm that baPWV closely tracks age-related increases in arterial stiffness and correlates with markers such as carotid intima-media thickness (CIMT) and left ventricular mass (12).

Notably, several studies have applied baPWV thresholds to identify individuals with subclinical vascular disease or increased atherosclerotic burden. In middle-aged hypertensive patients, baPWV was independently associated with CIMT, with an optimal cut-off of 18 m/s yielding an AUC of 0.77 for identifying increased CIMT (13). In a general male population, high baPWV (≥1,400 cm/s) was the only independent predictor of early carotid atherosclerosis after adjustment for age and cardiovascular risk factors, outperforming blood pressure (BP), dyslipidemia, and impaired fasting glucose (14). Furthermore, higher baPWV has been associated with a significantly greater prevalence of composite coronary and carotid atherosclerosis, demonstrating moderate discriminatory ability (AUC 0.692) for detecting individuals with concurrent multi-site atherosclerotic findings (15). Collectively, these findings indicate that arterial stiffness, as measured by baPWV, is mechanistically linked to structural atherosclerotic markers such as coronary artery calcium (CAC) scoring or CIMT, while remaining a distinct physiological process. Given this relationship and distinction, we defined the study outcome as subclinical arterial stiffness elevation (SASE) to reflect functional arterial stiffening rather than imaging-defined structural atherosclerosis.

Emerging evidence supports a shared physiological mechanism among GGT/HDL-C, baPWV, and CVD, particularly via pathways involving inflammation, insulin resistance, and endothelial dysfunction (5). Studies in asymptomatic individuals demonstrate that GGT is independently associated with SA, assessed by imaging and arterial stiffness indices (16), and suggest that GGT/HDL-C could serve as a superior potential risk marker compared to its individual components. Importantly, sex differences appear to modulate the predictive value of the GGT/HDL-C ratio. Women exhibit stronger associations between elevated GGT/HDL-C and cardiovascular risk, suggesting a potential role for hormonal or metabolic differences in disease progression (17).

Given these interconnections, the present study aims to explore the association between GGT/HDL-C and cardiovascular risk factors, baPWV, and sex-specific differences. Moreover, we aim to evaluate whether GGT/HDL-C is an independent potential marker of SASE and to identify a clinically relevant cut-off point that could aid early risk stratification and intervention.

## 2 Materials and methods

### 2.1 Study design and population

This cross-sectional observational study included adult participants aged ≥18 years who underwent annual health examinations at Xiamen Chang-Gung Hospital between 2013 and 2016. The study protocol was reviewed and approved by the Institutional Review Board of Xiamen Chang Gung Hospital and adhered to the ethical principles outlined in the Declaration of Helsinki.

The study data were not derived from a predefined research cohort, clinical trial, or funded longitudinal registry. Instead, the dataset consisted of routine annual self-paid health examination records collected in clinical practice. After IRB approval, de-identified digital records of eligible participants were retrieved and analyzed for research purposes. No prospective enrollment, intervention assignment, or scheduled follow-up was performed.

### 2.2 Inclusion and exclusion criteria

Eligible participants were required to have complete medical records, including documented past medical and medication histories. Individuals must have fasted for at least 12 h prior to their physical examination, and female participants were required to be non-pregnant at the time of assessment. The health examination included anthropometric measurements, BP, lipid profile, fasting plasma glucose, GGT, baPWV, and ankle-brachial index (ABI).

Exclusion criteria included individuals with chronic metabolic-affecting conditions such as thyroid dysfunction or chronic hepatitis, and those using hypoglycemic agents or corticosteroids at the time of evaluation.

### 2.3 Data collection and clinical measurements

Participants completed structured questionnaires regarding medical history and medication usage. Venous blood samples were collected by trained nursing staff following standardized operating procedures. All biochemical analyses were conducted in a hospital laboratory accredited by the College of American Pathologists (CAP). Fasting plasma glucose levels were measured using a modified hexokinase enzymatic assay (Cobas Mira Chemistry System, Roche Diagnostic Systems, Montclair, NJ, USA). Serum GGT, total cholesterol, LDL-C, HDL-C, and triglycerides were analyzed using a biochemical autoanalyzer (UniCel DxC 800 Synchron, Beckman Coulter, Ireland). All assays were performed using calibrated instruments to ensure accuracy.

BP was measured three times in a seated position after at least 15 min of rest using an automated sphygmomanometer. The mean arterial pressure (MAP) was calculated using the formula: MAP = (2/3 × DBP) + (1/3 × SBP). Height and weight were recorded to the nearest 0.1 cm and 0.1 kg, respectively, and body mass index (BMI) was computed as weight in kilograms divided by height in meters squared (kg/m^2^).

Alcohol intake was assessed using structured questionnaire data available from the health examination records. However, the questionnaire did not collect detailed quantitative information on alcohol intake, including grams of alcohol consumed per day or week, beverage type, duration of drinking, or binge drinking patterns. Therefore, alcohol intake could only be included as a categorical covariate in the regression models. Liver ultrasound or controlled attenuation parameter data were not available in the database; consequently, hepatic steatosis, non-alcoholic fatty liver disease (NAFLD), or metabolic dysfunction-associated steatotic liver disease (MASLD) could not be formally diagnosed or incorporated as covariates in the primary models.

### 2.4 Arterial stiffness assessment

baPWV and ABI were measured using a validated volume-plethysmographic oscillometric device (VP-1000; Colin Co. Ltd., Komaki, Japan). All measurements were performed by trained technicians according to a standardized protocol. After participants had rested in the supine position for at least five minutes in a quiet examination room, BP cuffs equipped with oscillometric and plethysmographic sensors were applied to both upper arms and both ankles and connected to the device. The VP-1000 system simultaneously recorded pulse volume waveforms and BP signals from all four limbs.

The pulse transit time between the brachial and ankle sites (Tba) was defined as the time interval between the initial upstroke, or wavefront, of the brachial waveform and that of the ankle waveform. The device automatically estimated the arterial path lengths using participant height-based equations. The estimated path length from the heart/suprasternal notch to the brachium was calculated as:

Lb = 0.2195 × height (cm)−2.0734

and the estimated path length from the heart/suprasternal notch to the ankle was calculated as:

La = 0.8129 × height (cm) + 12.328.

baPWV was then calculated using the following equation:

baPWV = (La−Lb)/Tba.

Bilateral baPWV values were obtained, and the mean of the left and right baPWV measurements was used for statistical analyses. ABI was calculated as the ratio of ankle systolic blood pressure (SBP) to brachial SBP. These measurement procedures and height-based estimation algorithms are consistent with validated VP-1000/form PWV-ABI protocols used in previous studies.

The VP-1000 system estimates baPWV noninvasively using simultaneous four-limb oscillometric waveform recordings. After height and demographic information are entered into the device, cuffs are placed around both upper arms and ankles. The device simultaneously records brachial and ankle pulse waveforms and determines the pulse transit time from the delay between the brachial and ankle waveform fronts. The arterial path length is not measured manually but is automatically estimated from body height using standardized regression equations. The difference between the estimated heart-to-ankle and heart-to-brachium path lengths is divided by the brachial–ankle pulse transit time to derive baPWV. This workflow reduces operator dependence and supports reproducible large-scale vascular assessment.

### 2.5 Definition of subclinical arterial stiffness elevation

SASE was defined as a mean baPWV greater than 1700cm/s, consistent with established thresholds for arterial stiffness and elevated cardiovascular risk (18).

### 2.6 Statistical analysis

Participants were first stratified by sex and age group (≤50 and >50 years). Within each age–sex subgroup, the GGT/HDL-C ratio was ranked in ascending order and categorized into tertiles. The lowest tertile (T1) was used as the reference category in logistic regression analyses. The exact subgroup-specific tertile thresholds are provided in Supplementary Table S1 to ensure reproducibility.

The distribution of continuous variables was assessed using the Shapiro–Wilk test. Normally distributed continuous variables are presented as mean ± standard deviation (SD), whereas non-normally distributed variables are summarized as median and interquartile range (IQR). Categorical variables are expressed as numbers and percentages.

For comparisons between two groups, categorical variables were analyzed using the chi-square test. Normally distributed continuous variables were compared using Student's t-test, whereas non-normally distributed variables were compared using the Mann–Whitney U test. For comparisons among three or more groups, categorical variables were analyzed using the chi-square test, normally distributed continuous variables were compared using one-way analysis of variance (ANOVA), and non-normally distributed variables were compared using the Kruskal–Wallis test.

The association between GGT/HDL-C tertiles and SASE was examined using hierarchical logistic regression models. Model 1, the base model, was adjusted for age, fasting glucose, triglycerides, and LDL-C. Model 2, the fully adjusted model, additionally included alcohol intake and lipid-lowering medication use. Results are presented as odds ratios (ORs) with 95% confidence intervals (CIs), using the lowest GGT/HDL-C tertile as the reference group. Sensitivity analyses were performed to assess the robustness of the primary associations. Specifically, logistic regression analyses were repeated after excluding participants with heavy alcohol intake and those receiving lipid-lowering medications. Heavy alcohol intake was defined according to available questionnaire information indicating frequent alcohol consumption, including drinking on ≥3–5 days per week or nearly every day. Variables with no within-subgroup variation were omitted from the corresponding regression model.

Receiver operating characteristic (ROC) curve analyses were performed to evaluate the discriminatory performance of the GGT/HDL-C ratio alone for identifying SASE. The area under the ROC curve (AUC) with 95% CIs was calculated separately for men and women. Optimal cut-off values were determined using the Youden index, defined as sensitivity + specificity−1, and the corresponding sensitivity and specificity were reported. Subgroup-specific ROC analyses were also performed separately for the four age–sex subgroups. To assess the incremental discriminatory value of GGT/HDL-C, a Base Model including age, fasting glucose, triglycerides, LDL-C, lipid-lowering medication use, and alcohol intake was compared with a Combined Model that additionally included the GGT/HDL-C ratio. ROC curves were generated separately by sex, and differences in AUCs were evaluated using bootstrap validation.

All analyses were conducted using SPSS version 26.0 (SPSS, Chicago, IL, USA). A two-sided *p*-value < 0.05 was considered statistically significant. 

### 2.7 Ethics approval and registration

The study protocol was reviewed and approved by the Institutional Review Board of Xiamen Chang-Gung Hospital (IRB No. XMCGIRB2022102) and was conducted in accordance with the principles of the Declaration of Helsinki. This study was based on de-identified data extracted from an existing routine self-paid health examination database and was designed as a retrospective cross-sectional observational analysis. It was not a clinical trial, interventional study, or prospectively registered longitudinal cohort. Therefore, clinical trial registration or clinical cohort registration was not applicable.

## 3 Results

### 3.1 Baseline characteristics of the study population

Baseline characteristics are shown in Table 1. A total of 10,518 participants were included in the analysis, comprising 5,877 men (55.9%) and 4,641 women (44.1%). The median age of the population was 46 years (IQR, 40–54 years). Compared with women, men had higher BMI, diastolic blood pressure (DBP), waist circumference, fasting glucose, triglycerides, GGT/HDL-C ratio, and baPWV. Conversely, women had higher HDL-C levels and lower GGT/HDL-C ratios than men. The overall prevalence of SASE, defined as mean baPWV >1700cm/s, was 7.6%, with no significant sex difference.

**Table 1:** Baseline characteristics of the study population by Sex.

Variable	Total (*n* = 10,518)	Men (*n* = 5,877)	Women (*n* = 4,641)	*P* value
Age, years	46 (40–54)	46 (40–54)	47 (40–55)	0.002^a^
BMI (kg/m^2^)	23.72 (21.53–25.96)	24.45 (22.39–26.49)	22.71 (20.72–24.98)	<0.001^a^
Mean arterial pressure (mmHg)	100.67 (91.00–112.00)	103.33 (94.33–113.67)	96.33 (87.00–108.67)	<0.001^a^
Waist circumference (cm)	83.0 (76.0–90.0)	87.0 (81.0–92.0)	78.0 (72.0–84.0)	<0.001^a^
Waist-to-height ratio	0.51 (0.47–0.54)	0.51 (0.48–0.54)	0.50 (0.46–0.54)	<0.001^a^
Fasting glucose (mmol/L)	5.06 (4.76–5.43)	5.11 (4.79–5.51)	4.99 (4.71–5.34)	<0.001^a^
Total cholesterol (mmol/L)	5.15 (4.54–5.81)	5.22 (4.61–5.86)	5.06 (4.46–5.74)	<0.001^a^
Triglycerides (mmol/L)	1.21 (0.84–1.82)	1.43 (1.00–2.12)	0.98 (0.72–1.41)	<0.001^a^
LDL-C (mmol/L)	3.27 (2.73–3.86)	3.38 (2.84–3.97)	3.12 (2.61–3.69)	<0.001^a^
GGT (U/L)	23.00 (15.00–39.00)	31.00 (21.00–51.60)	15.40 (12.00–22.90)	<0.001^a^
HDL-C (mmol/L)	1.28 (1.09–1.50)	1.17 (1.02–1.36)	1.42 (1.23–1.63)	<0.001^a^
GGT/HDL-C ratio	18.26 (10.78–33.33)	27.43 (17.16–45.79)	11.04 (7.99–17.07)	<0.001^a^
baPWV (cm/s)	1,291.50 (1,178.50–1,442.88)	1,317.00 (1,215.00–1,454.50)	1,251.00 (1,131.00–1,423.00)	<0.001^a^
ABI	1.12 (1.07–1.18)	1.14 (1.08–1.19)	1.11 (1.06–1.16)	<0.001^a^
SASE, *n* (%)	799 (7.60)	430 (7.32)	369 (7.95)	0.223^b^

BMI, body mass index; LDL-C, low-density lipoprotein cholesterol; GGT, gamma-glutamyl transferase; HDL-C, high-density lipoprotein cholesterol; baPWV, brachial-ankle pulse wave velocity; ABI, ankle-brachial index; SASE, subclinical arterial stiffness elevation.

Data are presented as median (interquartile range) or number (%).

a Continuous variables were assessed for normality using the Shapiro–Wilk test and compared using the Mann–Whitney U test.

b Categorical variables were compared using the chi-square test or Fisher's exact test, as appropriate.

### 3.2 Age-stratified associations between GGT/HDL-C and cardiometabolic parameters

Age-stratified characteristics according to GGT/HDL-C tertiles are presented in Tables 2A,B (men) and Tables 3A,B (women). Because tertile classification was performed separately within each sex–age subgroup, the numerical thresholds differed across subgroups. The exact GGT/HDL-C ranges defining T1, T2, and T3 for men ≤50 years, men >50 years, women ≤50 years, and women >50 years are shown in Supplementary Table S1.

**Table 2A:** Characteristics of the study population by age-specific GGT/HDL-C ratios in men ≤50 years.

Men≦50 years				
Variable	T1^a^	T2^a^	T3^a^	*P* value^b^
Number of subject	1,289	1,289	1,288	
GGT/HDL-C range	4.726–21.379	21.407–40.789	40.815–619.565	
Age, years	42 (38–46)	42 (38–46)	42 (38–46)	0.060
BMI (kg/m^2^)	22.70 (20.69–24.78)	24.91 (23.18–26.73)	25.95 (24.19–28.12)	<0.001
Mean arterial pressure (mmHg)	97.00 (90.33–106.33)	102.33 (94.33–111.00)	107.33 (98.00–117.67)	<0.001
Waist circumference (cm)	81.0 (76.0–87.0)	87.0 (83.0–92.0)	90.0 (85.0–95.0)	<0.001
Waist-to-height ratio	0.48 (0.45–0.51)	0.51 (0.49–0.54)	0.53 (0.50–0.56)	<0.001
Fasting glucose (mmol/L)	4.91 (4.66–5.21)	5.06 (4.76–5.39)	5.22 (4.90–5.68)	<0.001
Total cholesterol (mmol/L)	4.99 (4.47–5.57)	5.24 (4.66–5.79)	5.49 (4.84–6.19)	<0.001
Triglycerides (mmol/L)	1.02 (0.79–1.35)	1.54 (1.14–2.10)	2.18 (1.53–3.18)	<0.001
LDL-C (mmol/L)	3.20 (2.74–3.70)	3.50 (2.93–3.97)	3.53 (2.95–4.19)	<0.001
GGT (U/L)	19.00 (16.00–23.00)	33.00 (27.10–40.00)	71.00 (54.00–105.08)	<0.001
HDL-C (mmol/L)	1.32 (1.16–1.51)	1.13 (1.01–1.28)	1.09 (0.96–1.24)	<0.001
GGT/HDL-C ratio	15.00 (11.86–17.95)	29.08 (24.77–33.95)	62.77 (49.38–92.79)	<0.001
baPWV (cm/s)	1,255.00 (1,168.50–1,347.00)	1,276.50 (1,185.00–1,378.50)	1,319.75 (1,221.75–1,441.50)	<0.001
ABI	1.13 (1.08–1.18)	1.14 (1.08–1.19)	1.13 (1.08–1.19)	0.162
SASE	16 (1.24)	23 (1.78)	43 (3.34)	<0.001

GGT, gamma-glutamyl transferase; HDL-C, high-density lipoprotein cholesterol; BMI, body mass index; LDL-C, low-density lipoprotein cholesterol; baPWV, brachial-ankle pulse wave velocity; ABI, ankle-brachial index; SASE, subclinical arterial stiffness elevation.

Data are presented as median (interquartile range) or number (%).

a Tertiles were generated separately within each sex–age subgroup using the GGT/HDL-C ratio.

b Continuous variables were compared across tertiles using the Kruskal–Wallis test; categorical variables were compared using the chi-square test or Fisher's exact test, as appropriate.

**Table 2B:** Characteristics of the study population by age-specific GGT/HDL-C ratios in men >50 years.

Men > 50 years				
Variable	T1^a^	T2^a^	T3^a^	*P* value^b^
Number of subject	671	671	669	
GGT/HDL-C range	3.865–18.644	18.667–33.019	33.019–460.819	
Age, years	59 (55–64)	58 (54–63)	56 (53–61)	<0.001
BMI (kg/m^2^)	22.82 (20.58–24.44)	24.64 (22.91–26.38)	25.17 (23.33–26.93)	<0.001
Mean arterial pressure (mmHg)	101.67 (92.83–112.33)	106.33 (97.33–117.83)	109.67 (99.67–120.00)	<0.001
Waist circumference (cm)	82.0 (76.0–87.0)	88.0 (83.0–93.0)	90.0 (85.0–94.0)	<0.001
Waist-to-height ratio	0.49 (0.46–0.52)	0.52 (0.50–0.56)	0.53 (0.50–0.56)	<0.001
Fasting glucose (mmol/L)	5.07 (4.79–5.43)	5.21 (4.86–5.76)	5.41 (5.04–6.06)	<0.001
Total cholesterol (mmol/L)	5.14 (4.54–5.75)	5.16 (4.47–5.88)	5.32 (4.67–6.00)	<0.001
Triglycerides (mmol/L)	0.98 (0.74–1.28)	1.40 (1.04–1.96)	1.90 (1.32–2.71)	<0.001
LDL-C (mmol/L)	3.29 (2.81–3.83)	3.42 (2.85–4.08)	3.45 (2.81–4.05)	0.003
GGT (U/L)	17.20 (15.00–20.00)	28.00 (24.00–33.00)	55.00 (42.80–80.00)	<0.001
HDL-C (mmol/L)	1.32 (1.15–1.54)	1.11 (0.97–1.30)	1.08 (0.95–1.26)	<0.001
GGT/HDL-C ratio	13.84 (10.88–15.98)	25.00 (21.80–29.00)	48.84 (39.39–70.15)	<0.001
baPWV (cm/s)	1,378.00 (1,253.25–1,567.25)	1,420.00 (1,296.75–1,594.75)	1,463.50 (1,314.50–1,639.00)	<0.001
ABI	1.17 (1.12–1.22)	1.16 (1.10–1.21)	1.16 (1.10–1.21)	0.120
SASE	102 (15.20)	107 (15.95)	139 (20.78)	0.014

GGT, gamma-glutamyl transferase; HDL-C, high-density lipoprotein cholesterol; BMI, body mass index; LDL-C, low-density lipoprotein cholesterol; baPWV, brachial-ankle pulse wave velocity; ABI, ankle-brachial index; SASE, subclinical arterial stiffness elevation.

Data are presented as median (interquartile range) or number (%).

a Tertiles were generated separately within each sex–age subgroup using the GGT/HDL-C ratio.

b Continuous variables were compared across tertiles using the Kruskal–Wallis test; categorical variables were compared using the chi-square test or Fisher's exact test, as appropriate.

**Table 3A:** Characteristics of the study population by age-specific GGT/HDL-C ratios in women ≤50 years.

Women≦50 years				
Variable	T1^a^	T2^a^	T3^a^	*P* value^b^
Number of subject	944	944	941	
GGT/HDL-C range	2.714–8.205	8.209–12.418	12.429–330.612	
Age, years	41 (36–45)	41 (37–46)	43 (39–47)	<0.001
BMI (kg/m^2^)	20.93 (19.61–22.60)	21.82 (20.10–23.72)	23.43 (21.48–25.65)	<0.001
Mean arterial pressure (mmHg)	89.33 (83.00–96.33)	90.67 (84.00–99.00)	95.00 (87.00–105.00)	<0.001
Waist circumference (cm)	73.0 (68.0–78.0)	75.0 (70.0–80.0)	79.0 (74.0–85.0)	<0.001
Waist-to-height ratio	0.46 (0.43–0.49)	0.47 (0.44–0.51)	0.50 (0.47–0.54)	<0.001
Fasting glucose (mmol/L)	4.82 (4.60–5.05)	4.88 (4.62–5.15)	5.05 (4.78–5.36)	<0.001
Total cholesterol (mmol/L)	4.78 (4.31–5.36)	4.79 (4.26–5.31)	4.93 (4.33–5.59)	<0.001
Triglycerides (mmol/L)	0.71 (0.56–0.92)	0.85 (0.68–1.14)	1.18 (0.87–1.70)	<0.001
LDL-C (mmol/L)	2.77 (2.36–3.25)	2.91 (2.51–3.41)	3.12 (2.63–3.64)	<0.001
GGT (U/L)	11.00 (9.00–12.00)	14.00 (13.00–16.00)	24.00 (19.00–34.00)	<0.001
HDL-C (mmol/L)	1.63 (1.46–1.83)	1.42 (1.28–1.59)	1.28 (1.11–1.48)	<0.001
GGT/HDL-C ratio	6.63 (5.82–7.45)	9.93 (9.01–11.03)	18.25 (14.81–25.26)	<0.001
baPWV (cm/s)	1,135.75 (1,056.62–1,227.88)	1,162.50 (1,082.75–1,266.50)	1,216.50 (1,117.00–1,321.50)	<0.001
ABI	1.09 (1.04–1.14)	1.09 (1.04–1.15)	1.10 (1.04–1.15)	0.247
SASE	2 (0.21)	3 (0.32)	14 (1.49)	<0.001

GGT, gamma-glutamyl transferase; HDL-C, high-density lipoprotein cholesterol; BMI, body mass index; LDL-C, low-density lipoprotein cholesterol; baPWV, brachial-ankle pulse wave velocity; ABI, ankle-brachial index; SASE, subclinical arterial stiffness elevation.

Data are presented as median (interquartile range) or number (%).

a Tertiles were generated separately within each sex–age subgroup using the GGT/HDL-C ratio.

b Continuous variables were compared across tertiles using the Kruskal–Wallis test; categorical variables were compared using the chi-square test or Fisher's exact test, as appropriate.

**Table 3B:** Characteristics of the study population by age-specific GGT/HDL-C ratios in women >50 years.

Women > 50 years				
Variable	T1^a^	T2^a^	T3^a^	*P* value^b^
Number of subject	605	605	602	
GGT/HDL-C range	2.415–10.588	10.588–17.442	17.450–209.756	
Age, years	57 (53–61)	58 (54–62)	58 (54–62)	0.050
BMI (kg/m^2^)	22.56 (20.84–24.69)	24.19 (22.47–26.31)	25.07 (23.19–26.91)	<0.001
Mean arterial pressure (mmHg)	104.00 (93.33–114.67)	108.00 (97.00–119.67)	109.33 (99.08–119.92)	<0.001
Waist circumference (cm)	79.0 (74.0–84.0)	83.0 (78.0–88.0)	86.0 (80.1–90.0)	<0.001
Waist-to-height ratio	0.50 (0.47–0.54)	0.53 (0.50–0.57)	0.55 (0.52–0.58)	<0.001
Fasting glucose (mmol/L)	5.01 (4.74–5.31)	5.18 (4.90–5.63)	5.36 (4.98–6.04)	<0.001
Total cholesterol (mmol/L)	5.44 (4.87–6.16)	5.40 (4.77–6.04)	5.56 (4.90–6.19)	0.027
Triglycerides (mmol/L)	0.93 (0.72–1.17)	1.25 (0.92–1.66)	1.60 (1.18–2.25)	<0.001
LDL-C (mmol/L)	3.33 (2.89–3.86)	3.46 (2.93–4.02)	3.54 (2.99–4.15)	0.002
GGT (U/L)	12.30 (11.00–14.00)	18.00 (16.00–21.00)	33.00 (26.00–49.00)	<0.001
HDL-C (mmol/L)	1.56 (1.39–1.81)	1.35 (1.19–1.51)	1.23 (1.08–1.44)	<0.001
GGT/HDL-C ratio	8.15 (6.79–9.32)	13.24 (11.81–15.07)	26.24 (20.42–37.37)	<0.001
baPWV (cm/s)	1,376.00 (1,242.00–1,550.50)	1,456.00 (1,305.50–1,653.00)	1,477.50 (1,332.38–1,661.75)	<0.001
ABI	1.14 (1.09–1.18)	1.14 (1.09–1.19)	1.14 (1.09–1.19)	0.533
SASE	88 (14.55)	126 (20.83)	136 (22.59)	<0.001

GGT, gamma-glutamyl transferase; HDL-C, high-density lipoprotein cholesterol; BMI, body mass index; LDL-C, low-density lipoprotein cholesterol; baPWV, brachial-ankle pulse wave velocity; ABI, ankle-brachial index; SASE, subclinical arterial stiffness elevation.

Data are presented as median (interquartile range) or number (%).

a Tertiles were generated separately within each sex–age subgroup using the GGT/HDL-C ratio.

b Continuous variables were compared across tertiles using the Kruskal–Wallis test; categorical variables were compared using the chi-square test or Fisher's exact test, as appropriate.

In both younger (≤50 years) and older (>50 years) men and women, increasing GGT/HDL-C tertiles were generally associated with adverse cardiometabolic profiles, including higher BMI, BP, waist circumference, fasting glucose, triglycerides, and LDL-C. HDL-C decreased progressively across higher GGT/HDL-C tertiles.

**Figure 1:**
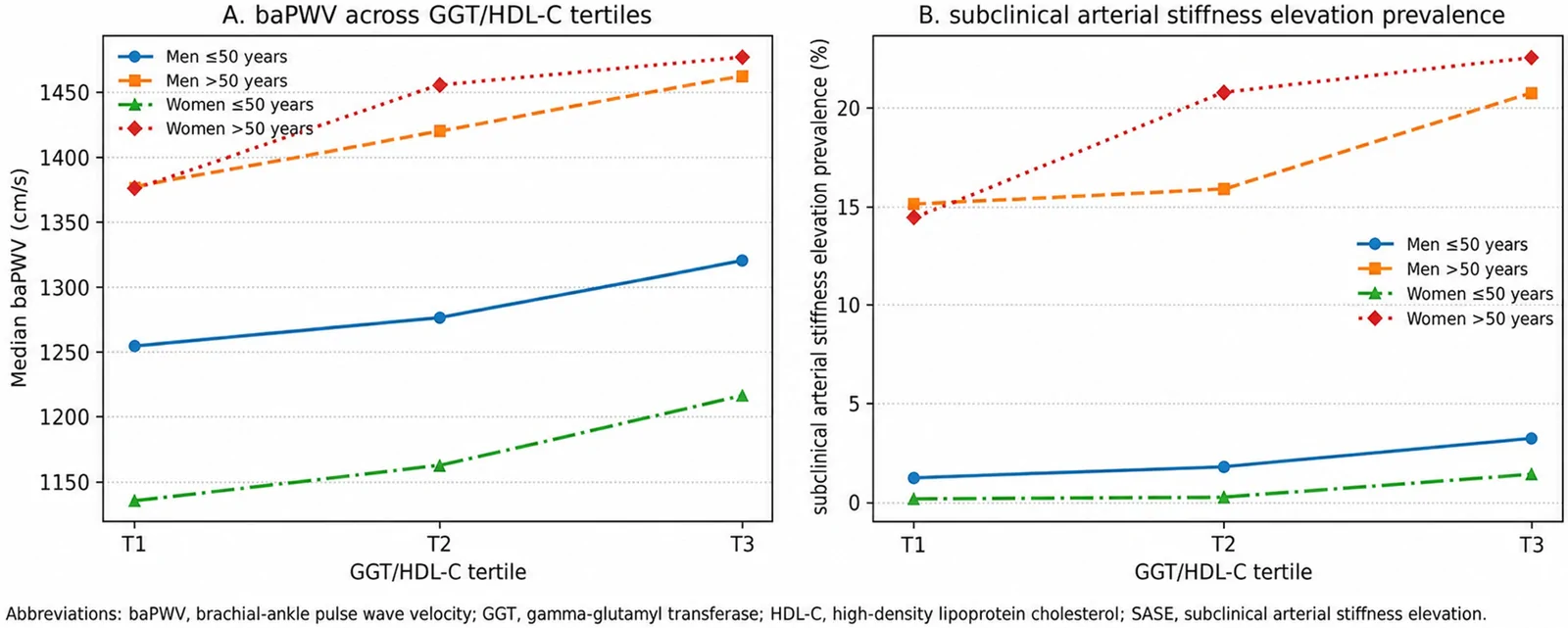
Trends in baPWV and subclinical arterial stiffness elevation prevalence across GGT/HDL-C tertiles by sex and age subgroup. Values are shown separately for men ≤50 years, men >50 years, women ≤50 years, and women >50 years. The figure illustrates the subgroup-specific distribution of brachial-ankle pulse wave velocity (baPWV) and the prevalence of subclinical arterial stiffness elevation across increasing tertiles of the GGT/HDL-C ratio. Subclinical arterial stiffness elevation was defined as mean baPWV >1700cm/s.

A significant increase in baPWV was observed across GGT/HDL-C tertiles in all four age–sex subgroups. Among younger individuals of both sexes, the rise in baPWV was particularly notable, suggesting increased arterial stiffness with elevated GGT/HDL-C ratios. To improve visualization of the sex- and age-specific findings, Figure 1 demonstrates that baPWV and the prevalence of SASE generally increased across ascending GGT/HDL-C tertiles, with subgroup-specific differences in the magnitude of these trends. The increase was visually more apparent in men ≤50 years and women >50 years, whereas the trend was relatively attenuated in men >50 years.

### 3.3 Association between GGT/HDL-C ratio and subclinical arterial stiffness elevation

**Figure 2:**
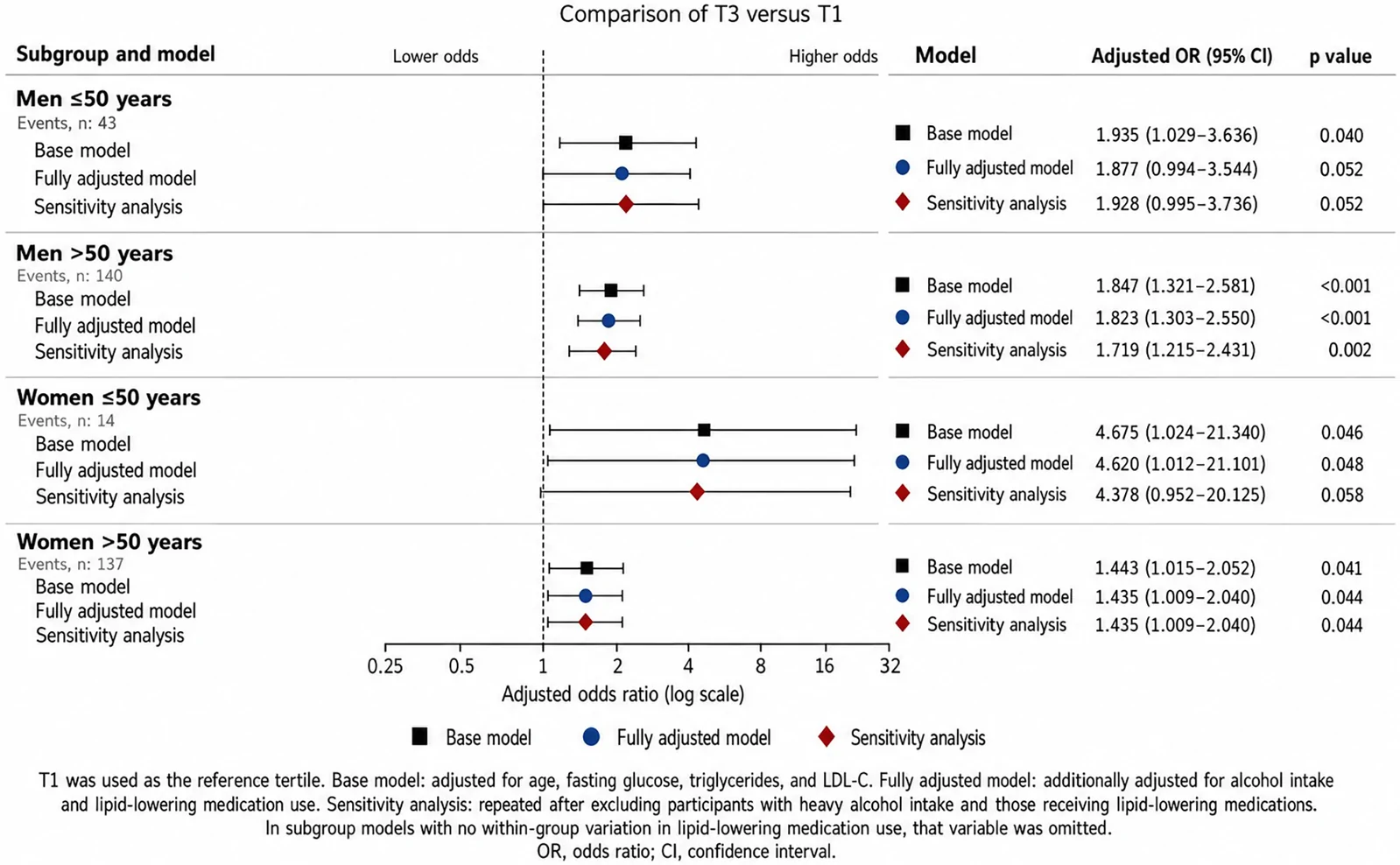
Forest plot of adjusted odds ratios for subclinical arterial stiffness elevation according to GGT/HDL-C tertiles. Odds ratios and 95% confidence intervals are shown for the highest versus lowest GGT/HDL-C tertile within each sex–age subgroup. The fully adjusted model included age, fasting glucose, triglycerides, LDL-C, alcohol intake, and lipid-lowering medication use. The forest plot provides a visual comparison of the magnitude and precision of subgroup-specific associations.

The association between GGT/HDL-C tertiles and SASE is summarized in Table 4. Two hierarchical logistic regression models were constructed. In the base model adjusted for age, fasting glucose, triglycerides, and LDL-C, the highest GGT/HDL-C tertile was associated with higher odds of SASE in men ≤50 years, men >50 years, women ≤50 years, and women >50 years. After additional adjustment for alcohol intake and lipid-lowering medication use, the association remained significant in men >50 years (OR, 1.823; 95% CI, 1.303–2.550), women ≤50 years (OR, 4.620; 95% CI, 1.012–21.101), and women >50 years (OR, 1.435; 95% CI, 1.009–2.040). In men aged ≤50 years, the estimate remained positive but did not reach conventional statistical significance in the fully adjusted model (OR, 1.877; 95% CI, 0.994–3.544). A forest plot (Figure 2) summarizes the odds ratios from the fully adjusted model for SASE comparing the highest with the lowest GGT/HDL-C tertile across the four age–sex subgroups. This visualization illustrates differences in the magnitude and precision of the associations across age–sex subgroups and complements the detailed estimates reported in the regression table.

**Table 4:** Hierarchical and sensitivity logistic regression models for subclinical arterial stiffness elevation according to GGT/HDL-C tertiles.

Comparison of the base model, fully adjusted model, and sensitivity analysis for T3 versus T1 of GGT/HDL-C ratio								
Group	Tertile	Events, n	base model OR (95% CI)^a^	*P* value	fully adjusted model OR (95% CI)^a^	*P* value2	Sensitivity analysis OR (95% CI)^b^^,^^c^	*P* value3
Men ≤50 years	T3 vs. T1	43	1.935 (1.029–3.636)	0.040	1.877 (0.994–3.544)	0.052	1.928 (0.995–3.736)	0.052
Men >50 years	T3 vs. T1	140	1.847 (1.321–2.581)	<0.001	1.823 (1.303–2.550)	<0.001	1.719 (1.215–2.431)	0.002
Women ≤50 years^d^	T3 vs. T1	14	4.675 (1.024–21.340)	0.046	4.620 (1.012–21.101)	0.048	4.378 (0.952–20.125)	0.058
Women >50 years	T3 vs. T1	137	1.443 (1.015–2.052)	0.041	1.435 (1.009–2.040)	0.044	1.435 (1.009–2.040)	0.044

Values are presented as odds ratios (ORs) and 95% confidence intervals (CIs) for subclinical arterial stiffness elevation comparing the highest GGT/HDL-C tertile (T3) with the lowest tertile (T1).

a The base model was adjusted for age, fasting glucose, triglycerides, and LDL-C. The fully adjusted model additionally included alcohol intake and lipid-lowering medication use.

b Sensitivity analyses were performed after excluding participants with heavy alcohol intake and those receiving lipid-lowering medications.

c In subgroup models where lipid-lowering medication use showed no within-group variation, this variable was omitted from model estimation.

d Results should be interpreted cautiously in women ≤50 years because of the small number of events.

Sensitivity analyses excluding participants with heavy alcohol intake and those receiving lipid-lowering medications yielded generally consistent results. The direction and magnitude of the associations were largely unchanged in men ≤50 years, men >50 years, and women >50 years. In women ≤50 years, the association was attenuated to borderline significance, with a wide confidence interval, likely reflecting the small number of events in this subgroup. These findings suggest that the primary associations were not materially driven by heavy alcohol intake or lipid-lowering medication use.

However, because alcohol exposure was not quantified by intake volume, drinking duration, or binge drinking pattern, and because liver ultrasound data were unavailable, residual confounding by alcohol-related liver injury or NAFLD/MASLD could not be excluded. Therefore, the observed associations should be interpreted as robust to available alcohol adjustment but not fully independent of hepatic steatosis or cumulative alcohol exposure.

### 3.4 ROC analysis of the GGT/HDL-C ratio

ROC analyses were performed to evaluate the discriminatory performance of GGT/HDL-C alone for SASE. In men, the GGT/HDL-C ratio demonstrated a modest discriminatory ability for identifying SASE, with an AUC of 0.541 (95% CI: 0.512–0.570, *p* < 0.001). The optimal cut-off value was 17.12, corresponding to a sensitivity of 81.2% and specificity of 25.2% (Youden index: 0.064).

In women, the discriminatory performance was comparatively stronger, with an AUC of 0.659 (95% CI: 0.627–0.690, *p* < 0.001). The optimal cut-off value was 11.5, yielding a sensitivity of 71.5% and specificity of 54.8% (Youden index: 0.263). These findings indicate relatively better discriminatory performance of GGT/HDL-C for SASE in women than in men.

**Figure 3:**
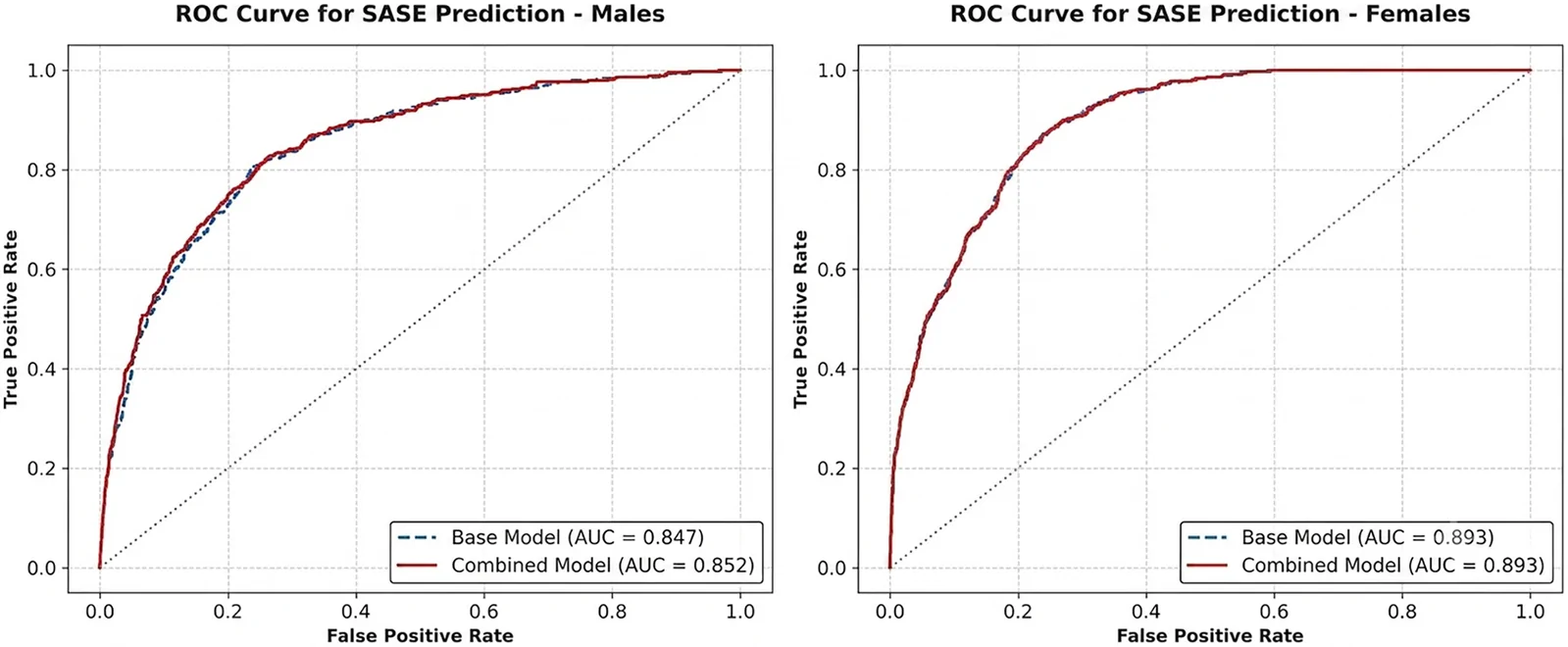
Comparison of receiver operating characteristic curves for the base and combined models for identifying subclinical arterial stiffness elevation. The “Base model” included the conventional covariates used in the fully adjusted model: age, fasting glucose, triglycerides, LDL-C, usage of lipid-lowering medication, and alcohol consumption. The “Combined Model” additionally included the GGT/HDL-C ratio. ROC curves were generated separately by sex, and the difference in the Area Under the Curve (*Δ*AUC) was calculated with bootstrap validation for statistical significance.

To evaluate the incremental discriminatory value of GGT/HDL-C, we compared a Base Model containing conventional covariates with a Combined Model that additionally included the GGT/HDL-C ratio. The comparative ROC curves are shown in Figure 3. The base model—comprising age, fasting glucose, triglycerides, LDL-C, lipid-lowering medication, and alcohol consumption—yielded an AUC of 0.847 in men. The addition of the GGT/HDL-C ratio (Combined Model) significantly improved the AUC to 0.852 (*Δ*AUC = 0.005, *p* = 0.024). In women, the base model demonstrated an initially high AUC of 0.893, and the addition of GGT/HDL-C maintained the AUC at 0.893 (*p* = 0.682), likely due to a ceiling effect in predictive accuracy by conventional factors.

Subgroup-specific ROC analyses are presented in Supplementary Table S2 and Supplementary Figure S1. The discriminatory performance of the GGT/HDL-C ratio differed across age–sex subgroups. The AUC was 0.642 (95% CI, 0.577–0.708) in men ≤50 years, 0.559 (95% CI, 0.525–0.593) in men >50 years, 0.696 (95% CI, 0.563–0.829) in women ≤50 years, and 0.576 (95% CI, 0.542–0.610) in women >50 years. The corresponding optimal cut-off values were 45.294, 39.904, 12.030, and 11.194, respectively. Although the highest AUC and Youden index were observed in women ≤50 years, this finding should be interpreted cautiously because of the limited number of events in this subgroup.

These cut-off values should be interpreted cautiously. Because they were derived from a single-center health check-up cohort in Southeast China, they should be considered exploratory thresholds for this specific population rather than broadly applicable clinical decision limits. External validation in independent multi-center cohorts is required before these cut-offs can be recommended for routine clinical screening or risk stratification.

## 4 Discussion

This study is among the first large-scale investigations to comprehensively examine the relationship between the GGT/HDL-C ratio and SASE, as assessed by baPWV, across sex and age strata. The uniqueness of our research lies in its focus on GGT/HDL-C as a potentially integrative biomarker of oxidative stress and lipid dysfunction and its application in predicting vascular health in asymptomatic adults. Several important findings emerged. First, we confirmed that higher GGT/HDL-C ratios are associated with adverse cardiometabolic profiles, including elevated BMI, BP, fasting glucose, and triglyceride levels. Second, individuals with higher GGT/HDL-C ratios generally exhibited higher baPWV and a greater prevalence of SASE. Third, the magnitude and statistical precision of the association between GGT/HDL-C and SASE varied across age–sex subgroups. In the fully adjusted model, the association remained statistically significant in men aged >50 years and in women of both age groups, whereas the estimate in men aged ≤50 years remained positive but did not reach conventional statistical significance. Sensitivity analyses yielded directionally consistent findings, although the association in women aged ≤50 years also became borderline significant because of the small number of events and wide confidence interval.

This study expands on existing literature by integrating GGT/HDL-C, a composite biomarker reflecting oxidative stress and lipid dysregulation, with baPWV, a validated index of vascular stiffness. Prior research has demonstrated that elevated GGT levels are associated with increased oxidative stress and arterial stiffness (19–21). Furthermore, GGT has been independently linked to subclinical coronary atherosclerosis, coronary artery stenosis severity, and adverse cardiac outcomes over long-term follow-up (6, 16), while HDL-C is known to exert anti-inflammatory, antioxidant, and endothelial-protective effects (8, 22). Our study confirms that the GGT/HDL-C ratio not only correlates with individual risk factors but may also serve as a unifying marker of cardiometabolic burden.

Notably, we observed a clear trend of increasing baPWV and SASE prevalence with higher GGT/HDL-C tertiles. This association persisted after adjusting for key confounders including age, glucose, triglycerides, and LDL-C. These findings are consistent with previous studies showing that lipid ratios such as TG/HDL-C and TC/HDL-C are predictive of arterial stiffness even in individuals with controlled LDL-C levels (23–25). They also reinforce the idea that vascular aging is influenced by oxidative and inflammatory processes beyond traditional lipid levels. While TG/HDL-C and non-HDL-C are widely used as surrogate markers of dyslipidemia and cardiovascular risk, GGT/HDL-C may offer additional value by incorporating hepatic oxidative burden. Recent research indicates that remnant cholesterol and triglycerides correlate more strongly with baPWV than LDL-C, even after adjustment for cardiovascular risk factors (26). Our findings complement this evidence by demonstrating that GGT/HDL-C similarly correlates with early vascular changes in apparently healthy individuals.

The ROC analysis suggested differences in the discriminatory performance of the GGT/HDL-C ratio for SASE between men and women. Although the overall discriminatory performance was modest, women showed a higher AUC and Youden index than men, indicating relatively better discrimination of SASE by GGT/HDL-C in women. This observation is consistent with previous studies reporting stronger cardiovascular associations of GGT-related biomarkers in women, potentially related to hormonal influences and postmenopausal metabolic changes. These findings suggest that the discriminatory performance of GGT/HDL-C may vary by sex and warrant further sex-specific validation. Nevertheless, the modest discriminatory performance and relatively low specificity, particularly in men, indicate that GGT/HDL-C should not be used as a standalone diagnostic or screening test.

Instead, the GGT/HDL-C ratio may serve as a practical, inexpensive adjunctive marker for identifying individuals who may be at higher risk of SASE, particularly when baPWV is not readily accessible, thereby complementing conventional cardiovascular assessments. This is especially relevant for asymptomatic individuals undergoing routine health evaluations, where early intervention could prevent the progression to overt cardiovascular disease. The determination of optimal GGT/HDL-C cut-off values in our population may help inform future risk-stratification studies and targeted preventive strategies.

Regarding our methodology, the selection of 1700cm/s to define SASE was driven by the specific clinical endpoints of interest. Prior studies indicate that optimal baPWV cut-off values vary according to the severity of the endpoint being assessed. Although lower thresholds (e.g., 1,413.5 cm/s) have been proposed for detecting early composite atherosclerotic changes (15), higher thresholds in the range of 1,700–1800cm/s have been associated with more advanced structural vascular changes, such as significant carotid intima-media thickening (13), and with elevated all-cause mortality (18). Although we defined our outcome as SASE to reflect functional stiffness, adopting this stringent threshold was intended to identify more pronounced vascular impairment that may be related to structural atherosclerotic burden, rather than merely benign physiological aging.

The sex- and age-specific patterns observed in this study may be explained by several interrelated biological mechanisms rather than by estrogen decline alone. First, GGT is not merely a hepatic enzyme or alcohol-related marker but is closely involved in extracellular glutathione metabolism. Under conditions of increased oxidative stress, elevated GGT activity may promote redox imbalance, facilitate LDL oxidation, and contribute to vascular inflammation and endothelial dysfunction. These processes are highly relevant to arterial stiffness, because reactive oxygen species reduce nitric oxide bioavailability, impair endothelial nitric oxide synthase signaling, increase vascular adhesion molecule expression, and promote vascular smooth muscle cell remodeling and extracellular matrix changes. Therefore, an elevated GGT/HDL-C ratio may reflect a combined pro-oxidative and lipid-related vascular risk phenotype that predisposes to increased baPWV and subclinical vascular injury (7).

Second, postmenopausal lipid remodeling may partly explain the association observed in women, particularly those aged >50 years. The menopausal transition is accompanied by adverse changes in lipid metabolism, including increases in total cholesterol, LDL-C, triglyceride-rich lipoproteins, and apolipoprotein B-containing particles (27). In addition, HDL-C concentration alone may not fully capture HDL functionality during midlife and menopause, because HDL particle composition and cholesterol efflux capacity may change across the menopausal transition. Therefore, a high GGT/HDL-C ratio in older women may represent not only reduced lipid-mediated vascular protection but also impaired antioxidant and anti-inflammatory HDL function in the presence of increased oxidative stress. This interpretation is consistent with recent longitudinal evidence showing that GGT/HDL-C was more strongly associated with incident cardiovascular disease in women than in men (7).

Third, visceral adiposity may interact with both GGT activity and postmenopausal lipid remodeling. In the present study, waist circumference and waist-to-height ratio increased progressively across GGT/HDL-C tertiles in both sexes, supporting the link between higher GGT/HDL-C and central adiposity. Visceral adipose tissue is metabolically active and promotes insulin resistance, chronic low-grade inflammation, oxidative stress, and perivascular adipose tissue dysfunction. These processes may accelerate endothelial dysfunction and arterial stiffening through increased cytokine release, reactive oxygen species generation, reduced nitric oxide bioavailability, and vascular smooth muscle cell proliferation. Menopause further promotes redistribution of adipose tissue toward visceral depots and is associated with adipocyte hypertrophy, immune cell infiltration, hypoxia, and fibrosis, providing a plausible mechanistic bridge between postmenopausal metabolic remodeling and increased arterial stiffness (28).

Preclinical findings also support this interpretation. In ovariectomized mouse models, estrogen deficiency has been shown to accelerate postmenopausal atherosclerosis and to be accompanied by increased lipid peroxidation and ferroptosis-related vascular injury, suggesting that estrogen loss may amplify oxidative lipid injury rather than acting as an isolated hormonal explanation. Accordingly, the association between GGT/HDL-C and SASE in women, especially older women, may reflect the convergence of oxidative stress, adverse lipid remodeling, central adiposity, and endothelial dysfunction. However, because the present study was cross-sectional and menopausal status was not directly measured, these mechanisms should be interpreted as biologically plausible explanations rather than causal evidence. Future studies incorporating direct measures of menopausal status, sex hormones, visceral fat imaging, inflammatory biomarkers, oxidative stress markers, and HDL functionality are warranted (29).

### 4.1 Strengths and limitations

The strengths of this study include its large sample size, standardized measurement protocols, and comprehensive assessment of both biochemical and vascular parameters in a middle-aged and older adult population. Additionally, the sex- and age-stratified analyses provide novel insight into sex-specific vascular aging patterns and the role of the GGT/HDL-C ratio in cardiovascular risk assessment.

The hierarchical modeling strategy and sensitivity analyses provide additional support for the robustness of the observed association between elevated GGT/HDL-C and SASE. The effect estimates were generally similar between the base and fully adjusted models, although the association in men aged ≤50 years no longer reached conventional statistical significance after additional adjustment. Furthermore, after excluding participants with heavy alcohol intake and those receiving lipid-lowering medications, the associations remained directionally consistent, particularly in men >50 years and women >50 years. These findings suggest that the observed associations were unlikely to be explained solely by these two potential confounders.

Several limitations should be acknowledged. First, this was a single-center study conducted at Xiamen Chang-Gung Hospital, and the participants were recruited from adults undergoing routine self-paid annual health examinations. This recruitment strategy may have introduced selection bias, because individuals who participate in health check-up programs may differ from the general population in health awareness, socioeconomic status, access to preventive healthcare, lifestyle behaviors, and underlying cardiometabolic risk profiles. Therefore, the study cohort may not fully represent community-dwelling adults, individuals with limited healthcare access, or patients encountered in routine primary care or cardiovascular clinics.

Second, the study population consisted exclusively of East Asian adults from Southeast China. Ethnic and geographic homogeneity may restrict the external validity of our findings. Differences in genetic background, dietary patterns, alcohol consumption, adiposity distribution, liver enzyme levels, HDL-C concentrations, cardiometabolic risk burden, and healthcare-seeking behaviors may influence both GGT/HDL-C levels and the prevalence of arterial stiffness. Accordingly, the observed sex- and age-specific associations between GGT/HDL-C and SASE should not be directly extrapolated to other ethnic groups or geographic populations without further validation.

Third, although ROC analyses identified optimal GGT/HDL-C cut-off values for SASE, these thresholds should be interpreted as exploratory and population-specific rather than definitive clinical cut-offs. As noted above, because these optimal cut-off values were derived from a single-center, ethnically homogeneous cohort, the cut-offs are applicable only to populations with similar demographic, ethnic, and clinical characteristics and should not be used as universal diagnostic or screening thresholds. Before clinical implementation, the proposed cut-off values require confirmation in large, multi-center, prospective external validation cohorts including participants from different regions, ethnic backgrounds, and healthcare settings.

Fourth, due to the absence of concurrent structural imaging (e.g., CIMT or CAC), we could not internally validate the baPWV threshold against imaging-defined structural atherosclerosis. We selected a relatively stringent threshold of 1,700cm/s, which has been associated with adverse clinical outcomes in prior East Asian studies (18). Nevertheless, baPWV reflects functional arterial stiffness and cannot substitute for direct structural imaging; therefore, the presence or burden of atherosclerosis could not be determined in the present study.

Fifth, alcohol intake and hepatic steatosis represent important sources of potential residual confounding in the present study. GGT is a sensitive but non-specific biomarker that may increase in response to alcohol exposure, cholestatic or hepatocellular injury, oxidative stress, and hepatic steatosis. Although alcohol intake was included in the fully adjusted model and sensitivity analyses were performed after excluding participants with heavy alcohol intake, the available questionnaire did not capture alcohol dose, drinking duration, beverage type, or binge drinking pattern. This may have resulted in exposure misclassification. If participants with higher GGT/HDL-C ratios also had greater unmeasured alcohol exposure, the association between GGT/HDL-C and SASE may have been partially overestimated. Conversely, nondifferential misclassification of alcohol intake could have attenuated the true association toward the null.

Sixth, the absence of liver ultrasound data is another major limitation. Because hepatic steatosis was not assessed, NAFLD/MASLD could not be formally diagnosed or adjusted for in the regression models. This is important because NAFLD/MASLD is closely associated with insulin resistance, visceral adiposity, dyslipidemia, systemic inflammation, endothelial dysfunction, and arterial stiffness. Recent clinical evidence indicates that fatty liver disease is linked to cardiovascular risk and vascular dysfunction, and studies have reported associations between NAFLD or MAFLD and increased arterial stiffness. Moreover, elevated GGT may serve as a surrogate marker of hepatic steatosis in non-drinkers, while the GGT/HDL-C ratio itself has recently been associated with NAFLD, hepatic steatosis severity, and liver fibrosis. Therefore, part of the observed association between GGT/HDL-C and SASE may reflect unmeasured fatty liver disease rather than a direct vascular effect of GGT/HDL-C alone.

From a mechanistic perspective, hepatic steatosis may act as both a confounder and an intermediate phenotype. It may confound the association because NAFLD/MASLD can increase GGT levels and independently contribute to arterial stiffness through metabolic inflammation and endothelial dysfunction. It may also lie on the causal pathway linking visceral adiposity, insulin resistance, oxidative stress, dyslipidemia, and vascular remodeling. Therefore, failure to account for hepatic steatosis may have influenced the magnitude of the observed associations. For this reason, the present findings should be interpreted as demonstrating an association between GGT/HDL-C and SASE in a health check-up population, rather than establishing GGT/HDL-C as a liver-independent vascular biomarker.

Finally, as the study was cross-sectional, causal inference cannot be established. Although GGT/HDL-C was associated with SASE after multivariable adjustment and sensitivity analyses, residual confounding from unmeasured or incompletely captured lifestyle, hepatic, renal, inflammatory, hormonal, and medication-related factors cannot be fully excluded.

Future longitudinal studies with more comprehensive clinical and lifestyle data are warranted to clarify the temporal relationship between GGT/HDL-C and vascular stiffness and to determine whether this biomarker improves risk prediction beyond conventional cardiovascular risk factors. Specifically, these future prospective studies should incorporate detailed alcohol assessment, including grams of alcohol per day or week, drinking duration, binge drinking pattern, and validated alcohol-use questionnaires. In addition, liver ultrasound, controlled attenuation parameter measurement, transient elastography, or other validated imaging-based assessments should be included to diagnose hepatic steatosis and liver fibrosis. Such data would allow stratified analyses by NAFLD/MASLD status and more rigorous evaluation of whether GGT/HDL-C predicts SASE independently of alcohol exposure and fatty liver disease.

## 5 Conclusion

In conclusion, this study demonstrates that the GGT/HDL-C ratio is independently associated with SASE in middle-aged and older adults, with distinct sex- and age-specific patterns. These findings suggest that GGT/HDL-C may serve as a practical and accessible adjunctive marker for preliminary vascular risk stratification, complementing conventional cardiovascular risk assessment rather than functioning as a standalone diagnostic tool. However, these findings should be interpreted cautiously because unmeasured alcohol exposure and hepatic steatosis may have influenced GGT levels and introduced residual confounding.

## Data Availability

The raw data supporting the conclusions of this article will be made available by the authors, without undue reservation.
